# A Phase II study of pulse dose imatinib mesylate and weekly paclitaxel in patients aged 70 and over with advanced non-small cell lung cancer

**DOI:** 10.1186/1471-2407-12-449

**Published:** 2012-10-03

**Authors:** Julie E Bauman, Keith D Eaton, Sarah G Wallace, Laurie L Carr, Sang-Joon Lee, Dennie V Jones, Hugo Arias-Pulido, Lisa A Cerilli, Renato G Martins

**Affiliations:** 1 Department of Internal Medicine, Divisions of Hematology/Oncology and Biostatistics, University of New Mexico Cancer Center, Albuquerque, New Mexico; 2 Department of Internal Medicine, Division of Medical Oncology, University of Washington, Seattle, WA, USA; 3 Division of Medical Oncology, National Jewish Health, Denver, CO, USA; 4 Department of Medicine, Division of Hematology/Oncology, University of Kentucky, Lexington, KY, USA; 5 Department of Pathology, University of Michigan, Ann Arbor, MI, USA; 6 University of Pittsburgh Cancer Institute, UPMC Cancer Pavilion, 5150 Centre Avenue, 5th floor, Pittsburgh, 15232, PA, USA; 7 Celltrion, Inc. Incheon, Korea

**Keywords:** Non-small cell lung cancer, Imatinib mesylate, Paclitaxel, Elderly, Interstitial fluid pressure, Platelet-derived growth factor, Frailty, Vulnerable elder survey

## Abstract

**Background:**

In non-small cell lung cancer (NSCLC), interstitial hypertension is a barrier to chemotherapy delivery, and is mediated by platelet derived growth factor receptor (PDGFR). Antagonizing PDGFR with imatinib may improve intra-tumoral delivery of paclitaxel, increasing response rate (RR).

**Methods:**

This single-stage, open-label phase II study evaluated pulse dose imatinib and weekly paclitaxel in elderly patients with advanced NSCLC. Eligible patients were aged ≥ 70 with untreated, stage IIIB-IV NSCLC and ECOG performance status 0-2. Primary endpoint was RR. Secondary endpoints included median progression free and overall survival (PFS, OS) and correlatives of PDGFR pathway activation. Baseline Charlson Comorbidity Index (CCI) and Vulnerable Elder Survey-13 (VES-13) were correlated with outcomes.

**Results:**

Thirty-four patients with median age 75 enrolled. Eleven of 29 (38%) were frail by VES-13 score. Overall RR was 11/34 (32%; 95% CI 17%-51%), meeting the primary endpoint. Median PFS and OS were 3.6 and 7.3 months, respectively. High tumoral PDGF-B expression predicted inferior PFS. Frail patients by VES-13 had significantly worse median PFS (3.2 vs. 4.5 months; p=0.02) and OS (4.8 vs. 12 months; p=0.02) than non-frail.

**Conclusions:**

The combination of imatinib and paclitaxel had encouraging activity as measured by the primary endpoint of RR. However, PFS and OS were typical for elderly patients treated with single agent chemotherapy and the regimen is not recommended for further study. Adjunct imatinib did not overcome the established association of tumoral PDGF-B expression with inferior PFS. VES-13 was a powerful predictor of poor survival outcomes. Frailty should be further studied as a predictor of non-benefit from chemotherapy.

**Trial Registration:**

ClinicalTrials.gov NCT01011075

## Background

Platelet-derived growth factor receptor (PDGFR) and its ligand, PDGF, constitute a tyrosine kinase signaling family involved in angiogenesis, inhibition of apoptosis, and regulation of interstitial fluid pressure (IFP) [1]. PDGF is a dimeric protein with 4 isoforms, which binds to the extracellular domain of two structurally related tyrosine kinase receptors, PDGFR-α and PDGFR-β. A classic target of PDGF is the stromal fibroblast which expresses both *α* and β receptors, predominantly β-type [2]. IFP in both normal and malignant tissues is actively regulated by fibroblast signaling through PDGFR-β. In solid tumors, elevated IFP is a barrier to delivery of chemotherapy, impeding transcapillary drug transport due to Starling forces [3]. Elevated IFP is caused by a dysfunctional stroma, featuring structurally abnormal capillaries and lymphatics, desmoplasia, and contraction of the interstitial matrix by fibroblasts [4]. The phenotype of interstitial hypertension is potentially reversible by PDGFR-β inhibition. Imatinib mesylate (Novartis; Basel, Switzerland) is a synthetic tyrosine kinase inhibitor targeting Bcr-Abl, c-Kit and PDGFR. In murine thyroid cancer xenografts, adjunct imatinib decreased IFP, increased uptake of epothilone B or paclitaxel, and increased anti-tumor effects relative to chemotherapy alone [5,6]. In non-small cell lung cancer (NSCLC) xenografts, imatinib decreased phosphorylated PDGFR-β, vascular endothelial growth factor, and IFP while increasing intratumoral delivery of docetaxel or liposomal doxorubicin [7].

Cytoplasmic expression of PDGF occurs in the majority of NSCLC and is a negative prognostic indicator, while PDGFR-β is expressed universally by tumor stroma [8-10]. Co-expression of PDGF and PDGFR-β raises the plausibility of a paracrine loop mediating interstitial hypertension and chemotherapy resistance. Elevated IFP up to 25 mmHg has been described in lung tumors, which may underlie low response rates to chemotherapy [11]. We hypothesized that antagonism of PDGFR-β with imatinib could increase the therapeutic index of weekly paclitaxel. Paclitaxel is a mitotic inhibitor which independently enhances perfusion and oxygenation, and decreases IFP [12,13]. Paclitaxel is superior to best supportive care in first line management of advanced NSCLC [14] and is indicated in combination with platinum for fit, age-unselected patients. A taxane is an accepted single agent standard in elderly patients with advanced NSCLC [15,16]. Here, we report the final results from a phase II clinical trial evaluating the combination of weekly paclitaxel and pulse dose imatinib in elderly patients with advanced, chemotherapy-naïve NSCLC.

## Methods

This multi-center study was approved by the institutional review boards of the University of Washington-Fred Hutchinson Cancer Research Center and the University of New Mexico. The clinical trial was publicly registered at ClinicalTrials.gov, NCT01011075. Key eligibility criteria included: age ≥ 70, diagnosis of advanced NSCLC (stage IIIB with pleural effusion or IV [17]); measurable disease according to modified RECIST criteria version 1.0 [18]; Eastern Cooperative Oncology Group performance status (ECOG-PS) 0 to 2; adequate organ function. Key exclusion criteria included: prior chemotherapy for advanced NSCLC; uncontrolled brain metastases; symptomatic neuropathy (Grade ≥ 2); serious or uncontrolled concomitant medical disorder. All patients provided written informed consent.

Patients were treated with up to six 28-day cycles of imatinib and paclitaxel. Paclitaxel 90 mg/m^2^ was administered intravenously on days 3, 10, and 17 of each 28-day cycle. Imatinib 600 mg daily was administered orally in 4-day pulses bracketing each paclitaxel infusion (days 1-4, 8-11, 15-18), adapted from the phase I design [19]. Pulse dose imatinib was selected based upon the theoretical mechanism of action of PDGFR-β blockade, pharmacokinetics of IFP response in xenografts, and inability to escalate paclitaxel with continuous imatinib in phase I. The dose limiting toxicity was neutropenia, consistent with earlier reports that continuous dose imatinib resulted in prohibitive hematologic toxicity when combined with cisplatin-irinotecan or gemcitabine [20,21].

The study incorporated a single-stage, open label, phase II design. An interim toxicity analysis was planned after the first 8 patients completed one cycle. The primary endpoint was response rate (RR) as measured by modified RECIST criteria version 1.0 [18]. The assumed null RR to single agent paclitaxel was 15%, as in CALGB 9730 [22], and a RR considered worthy of further study was 35%. Patients inevaluable for response were considered non-responders. A sample size of 35, with 33 eligible patients had 87% power to detect a true RR of 35%, and a 5% chance of falsely rejecting the null rate of 15%. The decision rule rejected the null hypothesis if ≥ 9 of 33 patients responded. Secondary efficacy endpoints included overall survival (OS), progression-free survival (PFS), and toxicity. Survival outcomes were analyzed by Kaplan-Meier methodology. Toxicity was described by National Cancer Institute Common Terminology Criteria for Adverse Events, version 3 [23]. Case report forms captured all grade ≥3 toxicities, any grade neuropathy or edema, and any grade event resulting in dose reduction or delay.

Exploratory objectives included measurement of tumor biomarker expression and assessment of patient comorbidity and frailty. Archived, formalin-fixed, paraffin-embedded (FFPE) tumor specimens were collected retrospectively for immunohistochemical (IHC) analysis. Staining for PDGF-B (Clone N-30; Santa Cruz Biotechnology, Inc., Santa Cruz, CA) and PDGFR-β (Clone Y92; Epitomics, Burlingame, CA) was performed by an optimized IHC staining protocol. Normal human placental tissue previously shown to be positive for PDGF-B and PDGFR-β was used as a positive control; the same tissue, incubated with an isotypic-matched antibody, was used as the negative control. Cytoplasmic PDGF-B and stromal PDGFR-β expression were graded using an H-score obtained by multiplying staining intensity (0 negative; 1+, weak; 2+, moderate; 3+, strong) by the percent of target cells with positive cytoplasmic or nuclear staining (0 to 100%) [24]. The study pathologist was blinded to outcome measures. Maximum likelihood estimates were conducted to describe the relationship of tumoral PDGF-B expression to RR, PFS and OS. The Vulnerable Elder Survey-13 [25] (VES-13) and Charlson Comorbidity Index [26] (CCI) respectively measured baseline frailty and comorbidity, to explore whether such measures could predict toxicity or survival outcomes. Frailty was defined as a VES-13 score of ≥ 3, the threshold associated with functional decline and mortality in the ambulatory, non-oncologic geriatric population [27]. We planned combined variable log-rank tests to determine whether a combination of VES-13, CCI, and/or ECOG-PS would perform better than a single variable in predicting toxicity or survival.

## Results

Thirty-four patients enrolled from September 2006 through April 2010 at three participating sites, including University of Washington, University of New Mexico and Puget Sound Oncology Consortium. Baseline patient characteristics are presented in Table 1. Median number of paclitaxel cycles was 2 (range 0 – 6). Nine patients (26%) required reduction of imatinib, with the most common reasons including neutropenia, neuropathy, and fatigue. Four patients (15%) required reduction of paclitaxel for neuropathy, elevated bilirubin, or fatigue.

**Table 1:** Baseline characteristics

**Characteristic**	**Number (%)**^**1**^
**Age (Years)**	
Median	74.5
Range	70-86
**Sex**	
Male	23 (68%)
Female	11 (32%)
**Histology**	
Adenocarcinoma	16 (47%)
Squamous	10 (29%)
Poorly differentiated	6 (18%)
Large cell/other	2 (6%)
**Stage**^**2**^	
IIIB	8 (24%)
IV	26 (76%)
**ECOG Performance Status (n=32)**	
0	10 (29%)
1	18 (53%)
2	4 (11%)
**Charlson Comorbidity Score (n=33)**	
Median	1
Range	0-7
**VES-13 Score (n=29)**	
Median	1
Range	0-8
VES ≥ 3 (Frail)	11 (38%)
**Tumor PDGF score (n=14)**^**3**^	
Median	75
Range	0-300

^1^Number(percent) unless units otherwise specified.

^2^American Joint Committee on Cancer, 6th ed.

^3^Score: Intensity of cytoplasmic staining x percent of tumor cells.

Treatment-emergent grade 3 or higher adverse events are summarized in Table 2. The most common grade ≥ 3 nonhematologic toxicities were fatigue, cardiac events, gastrointestinal events, infection, and rash. Cardiac adverse events included 2 episodes of grade 3 systolic dysfunction possibly related to imatinib, and 2 deaths from myocardial infarction and cardiac arrest attributed to pre-existing coronary artery disease. One death from infection and one from pneumonitis were considered protocol-related.

**Table 2:** Grade ≥ 3 adverse events

**Toxicity**	**Grade 3**	**Grade 4**	**Grade 5**	**Total**
**Hematologic**				7 (21%)
Anemia	1 (3%)	0	0	1 (3%)
Neutropenia	4 (12%)	1 (3%)	0	5 (15%)
Febrile Neutropenia	0	1 (3%)	0	1 (3%)
Thrombocytopenia	0	0	0	0
**Nonhematologic**				
Cardiac				4 (12%)
Systolic dysfunction	2 (6%)	0	0	2 (6%)
Cardiac arrest	0	0	1 (3%)	1 (3%)
Myocardial infarction	0	1 (3%)	1 (3%)	2 (6%)
Edema	1 (3%)	0	0	1 (3%)
Fatigue	10 (29%)	1 (3%)	0	11 (32%)
Infection	3 (9%)	0	1 (3%)	4 (12%)
Gastrointestinal				4 (12%)
Constipation	2 (6%)	0	0	2 (6%)
Diarrhea	2 (6%)	0	0	2 (6%)
Pulmonary				3 (9%)
Embolism	1 (3%)	0	0	1 (3%)
Pneumonitis	0	0	1 (3%)	1 (3%)
Pneumothorax	0	1 (3%)	0	1 (3%)
Bladder/Kidney stone	2 (6%)	0	0	2 (6%)

**Figure 1:**
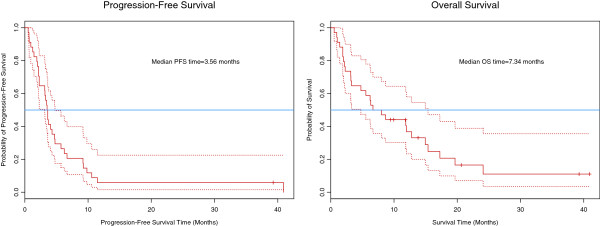
Progression-Free and Overall Survival.

Six patients were inevaluable for the primary endpoint, due to withdrawal or death prior to first response assessment. Per predefined intent-to-treat analysis, such patients were counted as non-responders. Eleven of 34 patients responded, with an overall RR of 32% (95% CI 17.4 – 50.5%), excluding the null rate of 15% and meeting the primary endpoint. Twelve patients had stable disease, with an overall disease control rate of 68%. Median PFS was 3.6 months, and median OS was 7.3 months (Figure 1).

**Figure 2:**
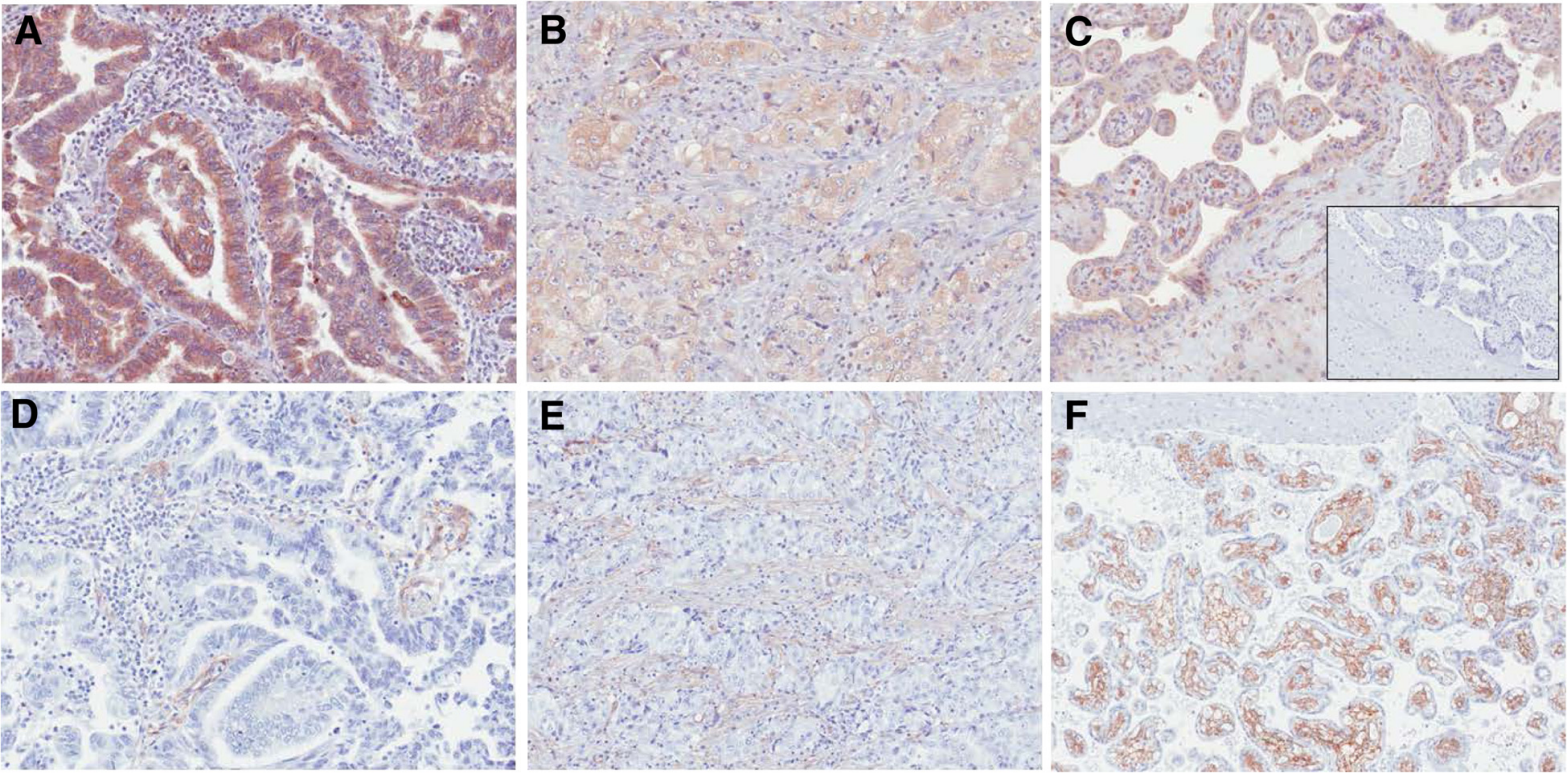
**PDGF-B and PDGFR-β Immunohistochemistry.** Legend: Representative immunohistochemical staining of PDGF-B (**A**-**C**) and PDGFR-β (D-F) in lung tumors (**A**, **B**, **D**, **E**) and human placenta (**C**, **F**). Inset shows the same placental tissue stained with an isotype-matched antibody.

Eighteen patients submitted archived tumor for correlative studies, and 14 specimens contained viable tumor for analysis of PDGF-B and PDGFR-β. Representative digital photomicrographs are presented in Figure 2. PDGF-B expression score was indirectly associated with PFS (p=0.03), with higher tumoral expression portending earlier progression. PDGF-B score was not associated with RR or OS. PDGFR-β was present universally in tumor stroma with variable intensity; no membranous or cytoplasmic staining was observed in epithelial cells. Stromal expression scores were not associated with RR, PFS or OS.

**Figure 3:**
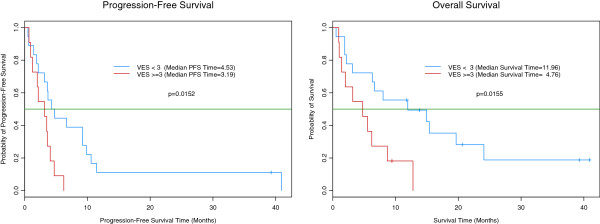
Progression-Free and Overall Survival According to VES-13 Frailty Score.

Measures of performance status, frailty and comorbidity did not predict RR. However, frailty was significantly associated with both PFS and OS (Figure 3). At baseline, 11 of 29 patients with available VES-13 scores met the definition for frailty. Only 3 frail patients had an ECOG-PS of 2; the remaining 8 had an ECOG-PS of 0 or 1. Frail patients had significantly worse median PFS (3.2 vs. 4.5 months; p=0.02) and OS (4.8 vs. 12 months; p=0.02) than non-frail. Frailty did not significantly predict toxicity. ECOG-PS was associated with OS, however not PFS or toxicity. Patients with ECOG-PS of 0 or 1 vs. 2 had median OS of 8.3 vs. 3.2 months (p=0.04). The CCI did not predict PFS, OS or toxicity. An exploratory, combined variable log rank test identified the combination of VES and ECOG-PS as the best predictor of differential PFS and OS. Here, good risk patients were defined as those with VES score < 3 (non-frail) and ECOG-PS of 0 or 1; the poor risk group included all others. Median PFS in the good vs. poor risk group was 4.5 vs. 3.2 months (p=0.01). Similarly, median OS was 12.0 versus 4.0 months (p=0.01).

## Discussion

In elderly patients with advanced NSCLC, first line treatment with the combination of intermittent, pulse dose imatinib and weekly paclitaxel demonstrated encouraging activity as measured by the primary endpoint of RR. As defined, the study fulfilled its primary endpoint; however two caveats warrant discussion. First, although RR was intriguing, both PFS and OS were numerically similar to values seen in the elderly population treated with single agent chemotherapy [28,29]. While the RR indicates that pulse dose imatinib may increase early anti-tumor effects from paclitaxel, the typical PFS and OS suggest that enhanced efficacy is not sustained. Second, several cautionary studies have now been published evaluating the combination of continuous dose imatinib and a related taxane, docetaxel, in solid tumors including NSCLC [30,31], breast cancer [32], androgen-independent prostate cancer [33], and ovarian cancer [34]. In these studies, patients were exposed to uninterrupted daily imatinib. These trials described poor tolerance; 7 of 8 were halted early for toxicity or lack of efficacy. Specifically in NSCLC, concern was raised for antagonism between continuous dose imatinib and docetaxel, due to a low observed RR [30].

Concurrent inhibition of PDGFR-β to overcome interstitial hypertension and improve tumoral delivery of chemotherapy has strong preclinical merit. Adjunct imatinib significantly decreased IFP and increased drug uptake in numerous solid tumor xenografts; efficacy measurably increased when considering apoptosis, proliferation rate, or tumor volume [5,6,35]. Imatinib may also serve an anti-angiogenic role, as PDGF-B signaling from endothelial cells maintains homeostasis of the pericyte. In tumor capillaries, pericytes have a structurally abnormal association with endothelial cells resulting in excess permeability, microaneurysms and impaired blood flow [36,37]. In tumor models, PDGF sequestration reduced pericyte coverage in tumor blood vessels; abnormal vessels were selectively pruned, normalizing the vascular phenotype [38,39]. In NSCLC xenografts, disruption of PDGF signaling similarly normalized tumor vasculature, enhancing uptake and efficacy of cyclophosphamide [40]. However, in the same model PDGF disruption without chemotherapy resulted in a wider perivascular sleeve of tumor cells, a higher index of tumor proliferation, and enhanced tumor growth. These paradoxical results caution that, while PDGFR-β blockade may be an effective adjunct to chemotherapy, alone it may be pro-proliferative due to improved circulatory efficiency. These preclinical findings may explain why the RR in our study stands in contrast to results from continuous dose imatinib and docetaxel in NSCLC. In our study, patients were exposed to 12 days of imatinib per 28-day cycle, limiting isolated PDGFR- β inhibition. In addition, pulse dose imatinib may have minimized proposed mechanisms of antagonism including arrest of tumor cells at the G0-G1 checkpoint by imatinib, or synergistic resistance mediated by each drug’s upregulation of p-glycoprotein [30]. Nonetheless, the hypothesized normalization of IFP and tumor vasculature by pulse dose imatinib improved only the efficiency and degree of response to paclitaxel, not the duration. Resistance to paclitaxel prevailed at the expected time point.

We hypothesized that patients with high intratumoral PDGF-B expression, an independent negative prognostic indicator in NSCLC [10], may differentially benefit from this strategy. Theoretically, tumoral production of PDGF-B may complete two pro-survival paracrine loops: PDGFR-β stimulation of stromal fibroblasts resulting in contraction of the extracellular matrix and elevated IFP; and PDGFR-β activation of neovascular pericytes. Upregulation of PDGF-B is a mechanism of chemotherapy resistance in glioma [41]. In our small sample, tumor PDGF-B expression was not associated with RR. However, higher PDGF-B expression scores were associated with reduced PFS. Because adjunct imatinib resulted in similar RR in patients with high or low expression of PDGF-B, disruption of PDGF signaling may have overcome intrinsic chemoresistance related to high IFP in PDGF-B overexpressing tumors. However, the strategy did not overcome the established association of high PDGF-B expression with poor PFS. We speculate that tumors with high PDGF-B expression may have a more phenotypically normal vasculature, secondary to mature pericyte coverage, limiting the anti-angiogenic benefit from adjunct imatinib.

The combination of pulse dose imatinib and weekly paclitaxel was adequately tolerated in this elderly cohort. There were 2 protocol-related deaths, numerically identical to single agent paclitaxel in this population [15]. The rate of treatment-emergent Grade ≥ 3 cardiac adverse events, which occurred in 4 patients (11.8%; 95% CI 3.3-27.5%), obligates additional discussion. This rate is numerically higher than reported for paclitaxel monotherapy in the elderly NSCLC population (5.7%; 95% CI 0.7-19.2%), although the confidence intervals overlap [15]. In our study, 2 patients had documented decline in systolic function, and 2 died from myocardial infarction in the context of pre-existing coronary artery disease, a prevalent comorbidity associated with increasing age in NSCLC [42]. Direct injury to the cardiomyocyte is a recognized toxicity of imatinib, consequent to c-Abl inhibition [43]. While causality cannot be ascribed to imatinib for cardiac events in this study, there is an established physiologic basis for potentiation of cardiac toxicity. Caution is justified should this combination undergo further development, particularly in patients with pre-existing heart disease.

We conducted baseline measures of comorbidity, frailty, and performance status, three components of comprehensive geriatric assessment, to determine associations with treatment vulnerability and survival. Medical comorbidity, the burden of chronic disease operationalized by the CCI, is associated with increased surgical complications and poor survival in NSCLC [43,44]. Frailty is the geriatric syndrome manifesting as reduced physiologic reserve and adaptivity to environmental stress. The VES-13 frailty score predicts mortality and hospitalization in ambulatory adults [27]. In oncology, frailty may predict treatment vulnerability better than chronologic age or disability [45,46]. ECOG-PS, a measure of cancer-related functional impairment, is prognostic in elderly patients with advanced NSCLC; moreover, an ECOG-PS of 2 is a better determinant of poor outcome than advanced age in patients receiving single agent docetaxel [47]. In our study, VES-13 was a powerful predictor of poor PFS and OS, and performed better than ECOG-PS which was associated only with OS. Moreover, VES-13 identified 8 vulnerable patients with a favorable ECOG-PS of 0 or 1. A combined variable log rank test distinguished 2 groups with an 8 month absolute difference in OS. Specifically, non-frail patients with ECOG-PS of 0 or 1 had a median survival of 12 months vs. 4 months in others. Thus, assessment of frailty with VES-13, a brief self-administered survey, adds valuable information in the selection of elderly NSCLC patients likely to experience survival benefit from chemotherapy.

This study has several important limitations. First, the absence of a randomized control group, exposed to single-agent paclitaxel and studied with identical biomarkers, particularly limits interpretation of our PDGF-B data. Second, while the majority of patients consented to optional tissue biomarkers, only 14 samples were analyzable, increasing the likelihood of Type II error. Third, the standard of care for unselected elderly patients with advanced NSCLC is evolving away from single agent chemotherapy. At design of this study, advanced age (>70) was a relevant selection criterion for single agent vs. platinum doublet chemotherapy, as addressed in national U.S. guidelines [48]. In the interval, an elderly-specific, randomized phase III trial documented superior survival from the combination of carboplatin-paclitaxel vs. gemcitabine or vinorelbine monotherapy [49]. Current guidelines emphasize patient selection by performance status rather than chronologic age [23]. Moreover, elderly patients are likely to benefit from molecular selection by epidermal growth factor receptor and ALK gene mutations.

## Conclusion

The regimen of pulse dose imatinib and weekly paclitaxel reached its primary endpoint, demonstrating an encouraging RR in elderly patients with advanced NSCLC. However, the benefit to adjunct imatinib was limited to response, without an associated improvement in PFS or OS. Reversal of elevated IFP and/or normalization of tumor vasculature may be most beneficial early in the course of cytotoxic chemotherapy. Given the standard of care for elderly, molecularly-unselected NSCLC patients has evolved to platinum doublet, as well as the significant question of cardiac toxicity, further development of this regimen does not appear justified. Frailty, as measured by the VES-13, is a powerful predictor of poor survival in elderly NSCLC patients and should be further studied as a predictor of non-benefit from cytotoxic chemotherapy.

## Abbreviations

(NSCLC): Non-small cell lung cancer; (PDGFR): Platelet-derived growth factor receptor; (RR): Response rate; (PFS): Progression free survival; (OS): Overall survival; (ECOG-PS): Eastern cooperative oncology group performance status; (CCI): Charlson comorbidity index; (VES-13): Vulnerable elder survey-13; (IFP): Interstitial fluid pressure; (FFPE): Formalin-fixed, paraffin-embedded; (IHC): Immunohistochemistry.

## Competing interests

Drs. Bauman and Martins acknowledge the research grant from the investigator-initiated trials program of Novartis, Inc. to the University of Washington for the conduct of this clinical trial. No competing interest is declared.

## Authors’ contributions

JB conceived and designed the study, treated study patients and drafted the manuscript; KE contributed to the design of the study and treated study patients; SW contributed to the design of the study and coordinated multi-institutional data collection; LLC and DJ treated study patients; SL contributed to the design of the study and conducted the statistical analysis; HP conducted IHC correlatives; LAC served as study pathologist; RGM contributed to the conception and design of the study and treated study patients. All authors revised the manuscript for critical intellectual content and approved the final manuscript.

## Pre-publication history

The pre-publication history for this paper can be accessed here:

http://www.biomedcentral.com/1471-2407/12/449/prepub
